# Cytokine Levels in Inner Ear Fluid of Young and Aged Mice as Molecular Biomarkers of Noise-Induced Hearing Loss

**DOI:** 10.3389/fneur.2019.00977

**Published:** 2019-09-11

**Authors:** Lukas D. Landegger, Sasa Vasilijic, Takeshi Fujita, Vitor Y. Soares, Richard Seist, Lei Xu, Konstantina M. Stankovic

**Affiliations:** ^1^Eaton Peabody Laboratories, Department of Otolaryngology, Massachusetts Eye and Ear, Boston, MA, United States; ^2^Department of Otolaryngology—Head and Neck Surgery, Harvard Medical School, Boston, MA, United States; ^3^Department of Otolaryngology, Vienna General Hospital, Medical University of Vienna, Vienna, Austria; ^4^Edwin L. Steele Laboratories, Department of Radiation Oncology, Harvard Medical School, Massachusetts General Hospital, Boston, MA, United States; ^5^Program in Speech and Hearing Bioscience and Technology, Harvard Medical School, Boston, MA, United States; ^6^Program in Therapeutic Science, Harvard Medical School, Boston, MA, United States

**Keywords:** perilymph, noise-induced hearing loss, cytokines, CXCL1, DARC, age-related hearing loss

## Abstract

Sensorineural hearing loss (SNHL) is the most common sensory deficit worldwide, frequently caused by noise trauma and aging, with inflammation being implicated in both pathologies. Here, we provide the first direct measurements of proinflammatory cytokines in inner ear fluid, perilymph, of adolescent and 2-year-old mice. The perilymph of adolescent mice exposed to the noise intensity resulting in permanent auditory threshold elevations had significantly increased levels of IL-6, TNF-α, and CXCL1 6 h after exposure, with CXCL1 levels being most elevated (19.3 ± 6.2 fold). We next provide the first immunohistochemical localization of CXCL1 in specific cochlear supporting cells, and its presumed receptor, Duffy antigen receptor for chemokines (DARC), in hair cells and spiral ganglion neurons. Our results demonstrate the feasibility of molecular diagnostics of SNHL using only 0.5 μL of perilymph, and motivate future sub-μL based diagnostics of human SNHL based on liquid biopsy of the inner ear to guide therapy, promote hearing protection, and monitor response to treatment.

## Introduction

Sensorineural hearing loss (SNHL) is the most common sensory deficit in the world. Disabling SNHL currently affects nearly half a billion people (1), and its incidence increases with age, affecting 16% of people aged 18 years and older, 34% of people aged 65–69 years, and 72% of people aged 85–90 years (2, 3). In addition to being economically costly to society, with the annual cost of unaddressed hearing loss being $750 billion globally (4), SNHL is physically and emotionally costly to individuals, as it is associated with cognitive dysfunction, dementia, and depression (5). Despite these staggering statistics, the cellular basis of human SNHL is typically unknown in living people because the inner ear cannot be biopsied today and its cells cannot be imaged clinically (6, 7). These barriers arise from the inner ear's small size, encasement in dense bone, and complex three-dimensional anatomy. There is a potential to overcome these barriers by performing liquid biopsy of the inner ear based on sampling of its fluid, perilymph, which bathes the vast majority of inner ear cells.

Noise exposure and aging are important causes of human SNHL, and noise exposure accelerates age-related hearing loss (8). According to the United States Department of Veteran Affairs, hearing loss and tinnitus are the most common disability claims of veterans (9). In addition to occupational noise exposure, recreational noise exposure is also threatening public health due to loud concerts, sporting events, and the increasing use of portable music players. The World Health Organization estimates that 1.1 billion young people are at risk of noise-induced hearing loss, primarily due to recreational exposure (4). Noise levels can cause permanent or temporary shifts in behavioral audiometric thresholds or physiologic metrics of hearing in both animals and humans (10). Permanent threshold shift (PTS) occur after exposure to high noise levels that cause irreversible structural damage in the cochlea, such as loss of hair cells, or their stereocilia and loss of cochlear neurons. Moderate noise exposure causes temporary threshold shift (TTS), characterized by a temporary elevation in physiologic or behavioral thresholds that recovers to pre-exposure levels within 1–2 weeks post exposure. A decade ago it was recognized that even TTS can cause permanent structural changes characterized by immediate loss of afferent synapses on inner hair cells, followed by a delayed loss of the associated neural somata (11). This neuropathic hearing loss is characterized by reduction in the amplitude of auditory brainstem response wave I, which reflects the activity of the cochlear nerve. However, not every TTS is associated with neuropathic hearing loss. Our laboratory has previously defined noise levels that differentiate neuropathic and non-neuropathic TTS in adolescent mice (12). In the current study we investigated how PTS, neuropathic TTS, and non-neuropathic TTS exposures are reflected in cytokine profiles in perilymph from young and old mice.

We focus on proinflammatory cytokines because they play an important role in a plethora of diseases (13), and are thought to be important mediators of numerous middle and inner ear maladies (14–17), including noise-induced hearing loss (18–21). However, past methods that have implicated cytokines in otologic disease are inappropriate for translation to living people, because they have required the destruction of sensorineural structures (e.g., for RT-PCR studies), post-mortem tissue processing (e.g., for immunohistochemistry), or pooling of samples from several animals to obtain an adequate volume of perilymph for analysis (22, 23).

While we (24, 25) and others (26–28) have demonstrated the feasibility of analyzing perilymph using proteomics or miRNA microarrays, cytokine levels in perilymph have never been directly measured before. Here we provide the first direct quantification of cytokine levels in perilymph and describe how these levels dynamically change after noise exposure and with aging. We demonstrate the feasibility of detecting molecular changes in as little as 0.5 μL of perilymph. We validate our findings by providing the first cellular distribution of CXCL1 and its putative receptor, DARC, in the cochlea. These results have important translational implications for future sub-μL based molecular diagnostics of human SNHL based on liquid biopsy of the inner ear to guide targeted therapy, promote hearing protection, and monitor patients' response to treatment.

## Results

### Cytokine Levels Are Increased in Perilymph After Exposure to PTS-Inducing Noise Levels

**Figure 1:**
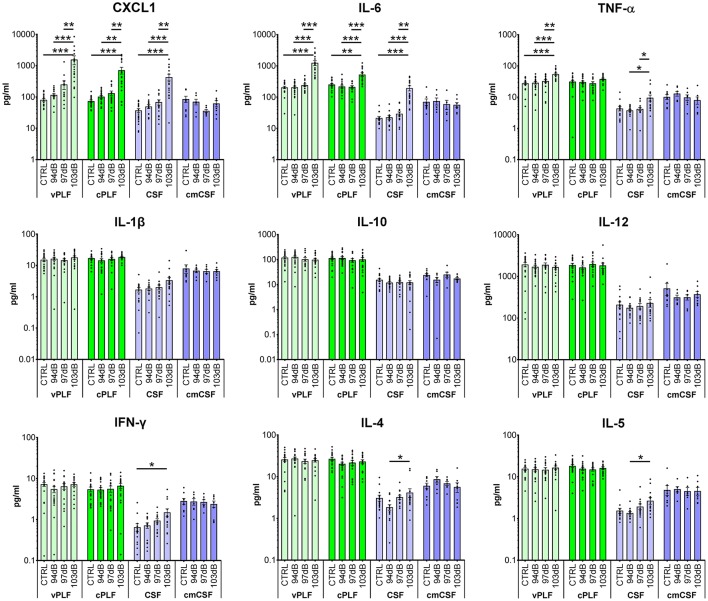
Cytokine levels in murine perilymph and cerebrospinal fluid 6 h after noise exposure. Six-week-old mice were exposed to 8–16 kHz noise for 2 h at 94 dB SPL (non-neuropathic TTS), 97 dB SPL (neuropathic TTS), and 103 dB SPL (PTS). Unexposed mice served as controls (CTRL). Six hours post exposure, vestibular perilymph (vPLF), cochlear perilymph (cPLF), and cerebrospinal fluid (CSF) were collected through the posterior semicircular canal. In addition, CSF was collected via cisterna magna (cmCSF). Each dot represents measurements from one ear (vPLF, cPLF, and CSF) or animal (cmCSF). Data are shown as group means ± standard error of the mean. *N* = 16–20 ears for vPLF, cPLF, and CSF, each; *N* = 14–17 ears for cmCSF; *N* = 7–10 ears for control samples. *P < 0.05, **P < 0.01, or ***P < 0.001.

Compared to the perilymph of unexposed animals (*N* = 19 ears), perilymph collected 6 h after exposing mice to noise levels that resulted in PTS (103 dB SPL at 8–16 kHz for 2 h) (*N* = 17 ears) demonstrated a statistically significant elevation of the levels of proinflammatory cytokines CXCL1 (vestibular perilymph = vPLF: 1,598.6 ± 509.0 *re* 83.0 ± 8.06 pg/mL, *p* < 0.001; cochlear perilymph = cPLF: 729.1 ± 178.6 *re* 75.8 ± 6.6 pg/mL, *p* = 0.001), IL-6 (vPLF: 1,258.9 ± 232.0 *re* 206.4 ± 19.6 pg/mL, *p* < 0.001; cPLF: 532.2 ± 72.2 *re* 254.8 ± 21.2 pg/mL, *p* = 0.0042), and TNF-α (vPLF: 56.2 ± 4.9 *re* 28.6 ± 2.7 pg/mL, *p* < 0.0001 (Figure 1). Of these 3 cytokines, CXCL1 exhibited the largest fold change: 19.3 ± 6.2 for vPLF and 9.6 ± 2.4 for cPLF (Supplementary Figure 1). Levels of IL-1β, IL-10, IL-12, IFN-γ, IL-4, and IL-5 did not significantly change 6 h after the same noise exposure. These results single out CXCL1 as the best candidate molecular biomarker of early PTS among the cytokines studied.

None of the tested cytokine levels demonstrated detectable changes 6 h after exposure to less intense noise that caused either non-neuropathic TTS (94 dB SPL at 8–16 kHz for 2 h) or neuropathic TTS (97 dB SPL at 8–16 kHz for 2 h) (Figure 1).

For cytokines with significant elevation after PTS-causing noise, the trends in cochlear and vestibular perilymph were generally similar, with vestibular perilymph showing higher inducible fluctuations than cochlear perilymph.

### Measured Cytokine Levels Are Significantly Higher in Perilymph Than in CSF

**Figure 2:**
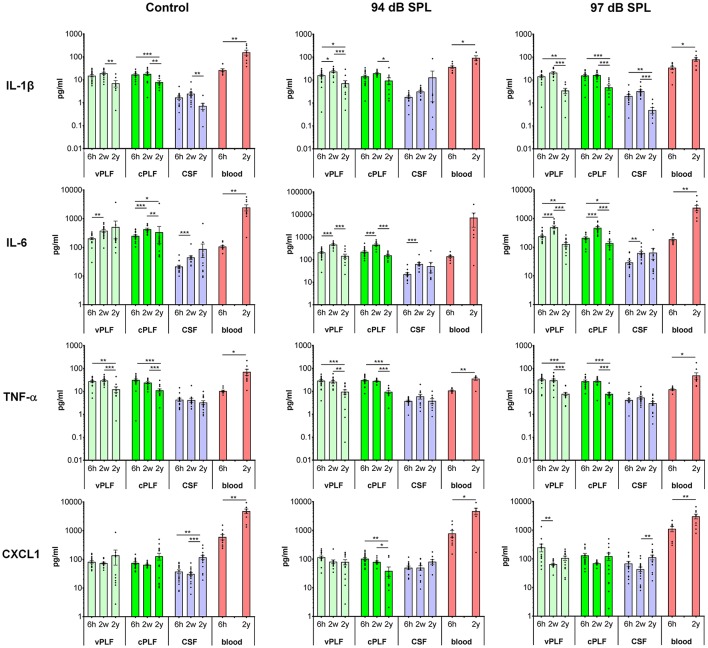
Levels of proinflammatory cytokines IL-1β, IL-6, TNF-α, and CXCL1 in murine perilymph, cerebrospinal fluid and blood 6 h, 2 weeks, and 2 years after noise exposure. Six-week-old mice were exposed to 8–16 kHz noise for 2 h at 94 dB SPL and 97 dB SPL; unexposed mice served as controls. Samples were collected 6 h, 2 weeks, and 2 years post exposure. Vestibular perilymph (vPLF), cochlear perilymph (cPLF), and cerebrospinal fluid (CSF) were collected through the posterior semicircular canal. In addition, blood samples were obtained by cardiac puncture after thoracotomy. Each dot represents measurements from one ear (vPLF, cPLF, and CSF) or animal (blood). Data are shown as group means ± standard error of the mean. *N* = 3–20 ears for vPLF; *N* = 3–20 ears for cPLF; *N* = 3–17 ears for CSF; *N* = 4–10 animals for blood; *N* = 7–10 ears for control samples. *P < 0.05, **P < 0.01, or ***P < 0.001.

The levels of all measured cytokines were higher in perilymph than in CSF (Figures 1, 2; Supplementary Figure 2). CSF obtained through the cisterna magna, abbreviated cmCSF (*N* = 10 ears), did not show significant changes in any of the measured cytokines, confirming the specificity of the cytokine reaction to the inner ear (Figure 1). However, CSF collected through the posterior semicircular canal (PSC) (*N* = 17 ears) did demonstrate statistically significant elevations in the levels of the same three cytokines that were elevated in perilymph 6 h after PTS-causing noise exposure (Figure 1). This indicates that CSF collected through the labyrinth is not identical to the CSF collected through the cisterna magna, as the former reflects noise-induced changes in the tissues it flows by. Nonetheless, CSF collected through the labyrinth can serve as a rough proxy for the CSF collected through the cisterna magna because both demonstrate lower cytokine levels than in perilymph.

### Levels of Proinflammatory Cytokines Demonstrate Dynamic Changes in Perilymph and Increase in Blood With Aging

The levels of proinflammatory cytokines IL-1β and TNF-α decreased significantly from age 8 weeks (corresponding to 2 weeks after noise exposure) to 2 years in control unexposed animals (vPLF: 19.80 *re* 7.18 pg/mL, *p* = 0.003; cPLF: 18.46 *re* 8.13 pg/mL, *p* = 0.0081 for IL-1β; vPLF: 30.78 *re* 12.61 pg/mL, *p* = 0.0010; cPLF: 24.79 *re* 11.48 pg/mL, *p* = 0.0009 for TNF-α; Figure 2). Similar significant age-related changes in perilymph were observed after non-neuropathic and neuropathic TTS exposure (Figure 2). Moderate upregulation of IL-1β was only observed in vPLF 2 weeks after non-neuropathic TTS exposure (*p* = 0.0420).

In contrast to IL-1β and TNF-α, IL-6 levels increased significantly at 8 weeks of age in all analyzed samples regardless of noise exposure (Figure 2). The observed difference in control animals could be explained by age-related developmental kinetics of immune-competent cells. While the number of T lymphocytes in mice plateaus at about 2–4 months of age (29), T-cell response in mice matures around 8 weeks of age (30). Similar developmental kinetics may exist for innate immune cells such as macrophages, which are an important source of IL-6 in the cochlear microenvironment. While the number of inner ear macrophages or their cytokine producing capacity have not been quantified between 6- and 8-week-old mice, the total number of macrophages in the small intestine is significantly increased between 4 and 9 weeks of age (31). At 2 years of age, IL-6 levels in vPLF and cPLF were significantly lower than at 2 weeks or 6 h after neuropathic noise exposure (vPLF: 130.00 at 2 years *re* 504.90 pg/mL at 2 weeks, *p* < 0.0001, and 242.72 pg/mL at 6 h post noise exposure; cPLF: 141.29 at 2 years *re* 482.23 pg/mL at 2 weeks, *p* = 0.0001, and 215.05 pg/mL at 6 h post noise exposure). This stands in contrast to the control animals, where IL-6 levels in vPLF did not meet our criterion for change between 2 weeks and 2 years after noise exposure.

The level of CXCL1 decreased significantly at 2 years compared to 6 h after exposure to non-neuropathic TTS in cPLF (*p* = 0.0033), and at 2 weeks compared to 6 h after exposure to neuropathic TTS in vPLF (*p* = 0.0147). In contrast, CXCL1 levels increased significantly in the blood of 2-year-old mice compared to 6-week-old mice regardless of noise exposure (unexposed controls: 4,895.74 *re* 616.11 pg/mL, *p* = 0.0039; 94 dB group: 4,827.11 *re* 797.90 pg/mL, *p* = 0.0256; 97 dB group: 3,087.72 *re* 1,141.61 pg/mL, *p* = 0.0093).

Similar to proinflammatory cytokines, T-helper cell (Th)-related cytokines (IFN-γ, IL-2, IL-4, IL-5), and immunoregulatory (IL-10 and IL-12) cytokines in the perilymph and CSF of 2-year-old animals showed decreased values compared to young animals, regardless of noise exposure (Supplementary Figure 2). However, 2 weeks after noise exposure, an increase in cytokine levels was observed in CSF for IL-2 (94 dB SPL), IL-4 (97 dB SPL), and IL-12 (97 dB SPL) compared to 6 h after noise exposure.

In blood samples, all analyzed proinflammatory cytokines increased significantly from 6 weeks to 2 years of age regardless of noise exposure (control group: *p* = 0.0092 for IL-1β, *p* = 0.0041 for IL-6, *p* < 0.0001 for TNF-α, *p* < 0.0001 for CXCL1; 94 dB SPL group: *p* = 0.0048 for IL-1β, *p* = 0.0360 for IL-6, *p* = 0.0076 for TNF-α, *p* = 0.0256 for CXCL1; 97 dB SPL group: *p* = 0.0127 for IL-1β, *p* < 0.0001 for IL-6, *p* < 0.0001 for TNF-α, *p* = 0.0093 for CXCL1; Figure 2). In the Th-related group of cytokines, Th1 cytokines (IFN-γ and IL-2) trended toward age-related increase in levels, whereas Th2 cytokines (IL-4 and IL-5) demonstrated decreased or unchanged levels in old animals when compared to young animals (Supplementary Figure 2). While IL-4 levels decreased significantly in old animals only in the control group (*p* = 0.0385), IL-5 levels were reduced in old animals of all groups (*p* = 0.0041 in the control group, *p* = 0.0176 in the 94 dB SPL group, *p* < 0.0001 in the 97 dB SPL group). The blood levels of immunoregulatory cytokine IL-10 were increased in old compared to young animals (*p* = 0.0027 in the control group, *p* = 0.0330 in the 94 dB SPL group, *p* < 0.0001 in the 97 dB SPL group). In contrast, blood IL-12 levels were similar between young and old animals, regardless of noise exposure (Supplementary Figure 2).

### CXCL1 Localizes to Specific Cochlear Supporting Cells

**Figure 3:**
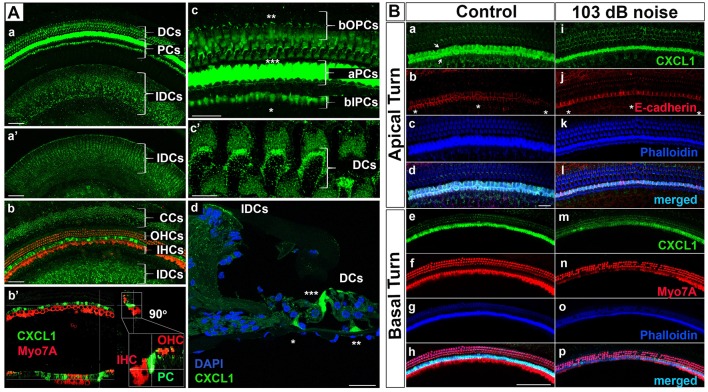
CXCL1 immunoreactivity localizes to cochlear supporting cells and is reduced in pillar cells after noise exposure. **(A)** Cochlear whole mounts from 6-week-old control mice **(Aa–c'; Ba–h)** and age-matched mice exposed to noise causing PTS **(Ad; Bi–p)** revealed immunostaining for CXCL1 (green) in specific supporting cells but not Myo7A-expressing hair cells (red) **(Ab-b')**. **(Ad)** is a paraffin cross section. CXCL1 is expressed in Claudius cells (CCs) **(Ab)**, Deiters cells (DCs) **(Aa,c',d)**, pillar cells (PCs) **(Aa,b-b',c,d)**, and interdental cells (IDCs) **(Aa,b)**. CXCL1 is predominantly expressed in the apical part of PCs (aPCs) **(Ac,d)** and to a lesser extent in the basilar part of inner PCs (bIPCs) and outer PCs (bOPCs). Pillar cells are positioned between outer (OHCs) and inner hear cells (IHCs) **(Ab-b')**. Asterisks indicate bIPCs (*), bOPCs (**), and aPCs (***) **(Ac,d)**. A magnified view of DCs shows CXCL1 positivity in the phalangeal processes of DCs **(Ac')**. A selected section from a z-stack illustrates CXCL1 positivity in IDCs **(a')**. CXCL1 is also expressed in the apical surface of IDCs **(Aa,b,d)**. Slides represent maximum projections **(Aa,b,c-c')** and orthogonal sections **(Ab')** of confocal z-stacks through whole mounts of the organ of Corti in the cochlear basal region (~35 kHz). Scale bars: 5 μm **(Ac')**, 25 μm **(Ac,d)**, 50 μm **(Aa,b)**. **(B)** Cochlear whole mounts immunostained with anti-CXCL1 antibody (green) **(Ba,e,i,m)**, anti-E-cadherin antibody to identify pillar cells (red) **(Bb,j)**, anti-Myo7A to identify hair cells (red) **(Bf,n)**, and Phalloidin (blue) **(Bc,g,k,o)** revealed CXCL1 and E-cadherin co-expression in pillar cells **(Bb,j)**. The asterisk indicates the modiolar side of the organ of Corti **(Bb,j)**. Arrows indicate inner and outer pillar cells **(a)**. The number of CXCL1-positive cells was significantly reduced only in the cochlear base of noise-exposed animals **(Bm)** where OHC loss was noted. Scale bars: 20 μm (B-apical turn), 100 μm (B-basal turn). The slides represent maximum projections of confocal z-stacks through cochlear whole mounts in the apical and basal region of the cochlea (~8 and 32 kHz region, respectively). Representative images are based on biological replicates in 4 control and 6 noise-exposed animals.

**Figure 4:**
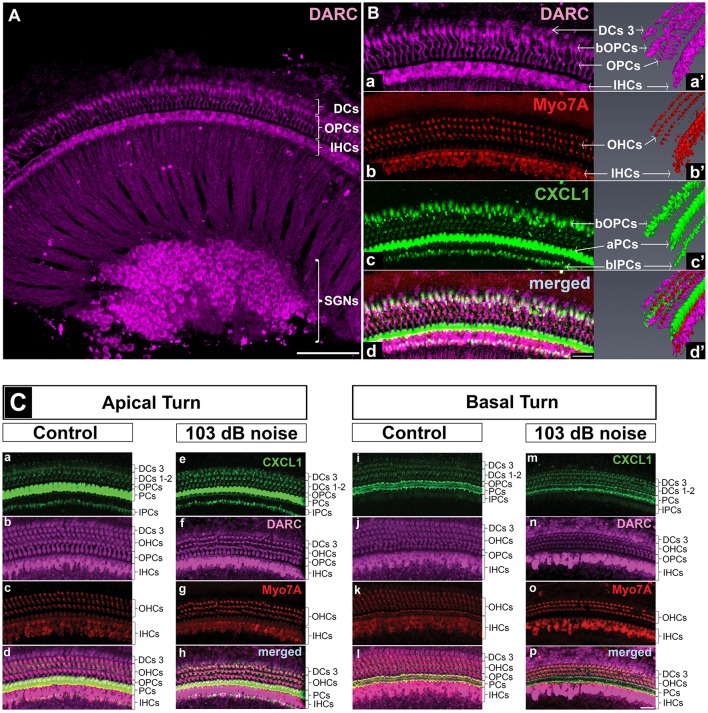
DARC, atypical chemokine receptor, is expressed in cochlear hair cells, supporting cells, and spiral ganglion neurons. Cochlear whole mounts of control mice **(A,B,Ca-d,i-l)** and age-matched mice exposed to PTS-causing noise (103 dB SPL) **(Ce–h,m–p)** at 6 weeks of age and sacrificed 6 h later were stained with anti-DARC (magenta), anti-CXCL1 (green), and anti-Myo7A antibody (red). **(A)** DARC is brightly expressed in the outer pillar cells (OPCs), inner hair cells (IHCs) and spiral ganglion neurons (SGNs), and moderately expressed in outer hair cells (OHCs) and Deiters cells (DCs). Scale bar, 100 μm. **(B)** A high magnification view of the whole mount shown in A and a corresponding 3D reconstruction illustrate DARC expression in the columnar part of OPCs and DARC coexpression with CXCL1 in the basilar part of OPCs (bOPCs) **(Ba–d,a'–d')**. Three-dimensional reconstruction of CXCL1 expression confirms the finding illustrated in the Figure 5, highlighting the main localization of CXCL1 in the apical part of PCs (aPCs) followed by the basilar parts of inner PCs (bIPCs) and outer PCs (bOPCs) **(Bc')**. Scale bar, 25 μm. **(C)** PTS-causing acoustic trauma did not appreciably affect DARC positivity while causing reduction in CXCL1 positivity in the basal turn of PCs. Scale bar, 25 μm. The slides represent maximum projections of confocal z-stacks through whole mounts in the apical and basal region of the cochlea (~8 and 32 kHz region, respectively). Representative images are based on biological replicates in 4 control and 3 noise-exposed animals.

While cellular expression of pro-inflammatory cytokines TNF-α and IL-6 has been previously reported in cochlear tissue (18), the cellular pattern of CXCL1 expression remains unknown. Therefore, we used immunohistochemistry applied to cochlear whole mounts of 6-week-old mice (*N* = 4 animals) to identify CXCL1-positive cells (Figure 3). The strongest immunoreactivity was observed in the cells adjacent to hair cells (Figure 3Aa). Co-staining for a hair-cell marker, Myo7A, revealed the strongest CXCL1-immunoreactivity between inner and outer hair cells, corresponding to the location of pillar cells (Figure 3Ab–b'). Further co-staining for a pillar cell marker, E-cadherin, as well as phalloidin, confirmed the pillar cells to be expressing CXCL1 at the highest level (Figure 3Ba,b). Using a comparative analysis of cochlear cross sections, whole mounts and 3D reconstructions, we observed that CXCL1 was differently expressed in the apical and basilar part of pillar cells, with predominant localization in the apical segment (Figure 3Ac,d). Basilar localization of CXCL1 in pillar cells was more prominent in the cochlear apex, suggesting a possible tonotopic distribution of CXCL1 (Figure 4Ca,i).

In addition to the dominant expression of CXCL1 in pillar cells, a marked CXCL1 positivity was found between outer hair cells. High magnification imaging (Figure 3Ac') and Myo7A co-staining (Figures 3Ab-b',Bh) identified CXCL1 expression in the phalangeal processes of Deiters cells. Additionally, CXCL1 immunoreactivity was detected in interdental cells (Figure 3Aa–a') and Claudius cells (Figure 3Ab). CXCL1 expression in these cells was also confirmed by the immunostaining of paraffin cross sections (Figure 3Ad). A semi-quantitative comparison of the staining intensity of CXCL1-positive cells is provided in Supplementary Table 1.

### Noise Trauma Reduces CXCL1 Immunoreactivity in Pillar Cells

Considering the pivotal role of CXCL1 in the development of the inflammatory response, along with our finding that among the analyzed proinflammatory cytokines in perilymph CXCL1 experienced the largest noise-induced elevation, we studied the effect of acoustic trauma on CXCL1 immunoreactivity in pillar cells. Immunostaining of cochlear whole mounts from control (*N* = 4 animals) and noise-traumatized mice sacrificed 6 h after PTS-causing noise exposure (*N* = 6 animals) showed a noticeable decrease in CXCL1 immunoreactivity in pillar cells. The reduced CXCL1 expression was detectable in the cochlear base (Figure 3Bm) but not the apex (Figure 3Bi), as evident in the representative cochlear whole mounts. This noise exposure also caused the loss of outer hair cells (Figure 3Bn,o,p), consistent with previous reports (32). The number of outer hair cells was dramatically and significantly reduced at and above 31 kHz while the number of inner hair cells remained intact along the cochlear length (Supplementary Figure 3).

In order to identify other possible sources of CXCL1 in inflamed cochlear tissue, we compared the distribution of Iba-1 and CXCL1 positive cells in the cochlea because fibrocytes and Iba1-positive cochlear macrophages have the capacity to participate in the cochlear immune response after acoustic overstimulation (18, 33). Immunohistochemical analysis of cochlear sections (*N* = 3 control animals and *N* = 3 noise-exposed animals) revealed that the cochlear cells expressing Iba1 did not co-express CXCL1 (Supplementary Figures 4B,C). Instead, Iba1-negative pillar cells that were CXCL1-positive in control mice (Supplementary Figure 4A) continued to express CXCL1 6 h after exposure to PTS-causing noise that targets the cochlear base (8–16 kHz noise at 103 dB for 2 h, Supplementary Figure 4B).

### DARC, a CXCL1 Receptor, Is Expressed in Cochlear Hair Cells, Supporting Cells and Spiral Ganglion Neurons

To identify the receptors through which CXCL1 may be exerting its effect in the inner ear, we studied expression of CXCR2 and DARC. We did not detect CXCR2 expression in the mature inner ear by qRT-PCR or immunostaining, consistent with a prior report (18).

In contrast to CXCR2, we saw specific immunostaining for DARC in the hair cells, outer pillar cells, a third row of Deiters cells, and spiral ganglion neurons (*N* = 4 animals) (Figure 4A). The analysis of DARC and Myo7A co-expression showed that DARC was more highly expressed in inner hair cells than in outer hair cells (Figures 4A,Ba,d,Cb,f,j,n). Considering the potential role of DARC in binding of CXCL1, we analyzed their co-expression and found that DARC was expressed in the columnar and basilar part of outer pillar cells, with CXCL1 co-localization only in the basilar part (Figure 4Ba,a',d,d'). In contrast to CXCL1, whose expression was reduced in the cochlear basal turn after exposure to noise targeting the base (Figure 4Cm), DARC immunoreactivity was not noticeably affected by noise exposure (*N* = 4 animals) (Figure 4Cb,f,j,n). A semi-quantitative comparison of the staining intensity of DARC-positive cells is provided in Supplementary Table 2.

**Figure 5:**
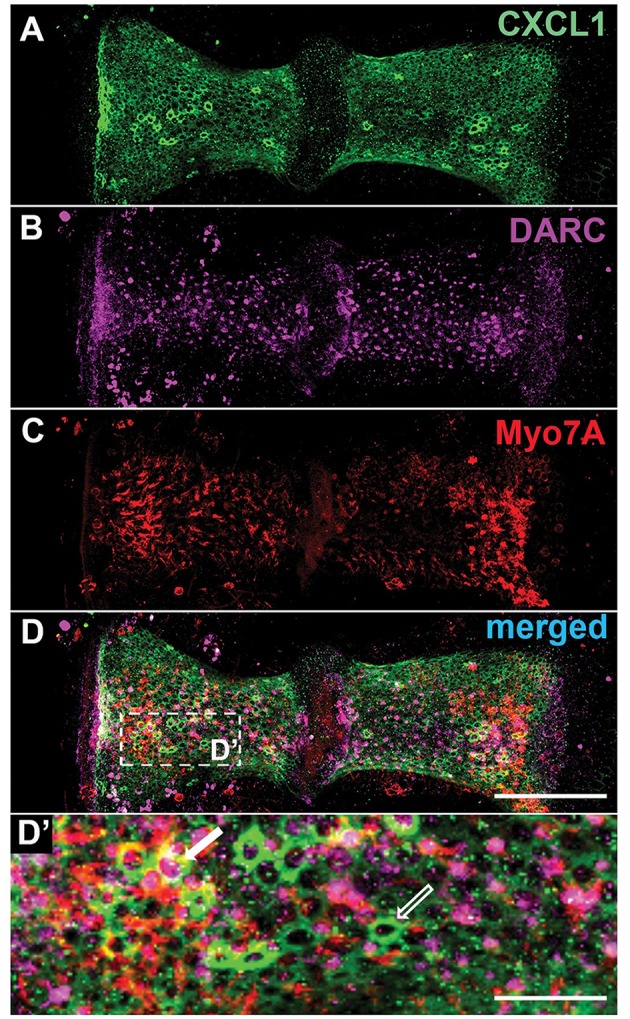
Within the crista ampullaris, CXCL1 localizes to supporting cells while DARC preferentially localizes to a subset of hair cells. Whole mounts of the crista ampullaris of control mice stained with anti-CXCL1 (green) **(A)**, anti-DARC (magenta) **(B)**, and anti-Myo7A (red) **(C)** antibodies. CXCL1 is expressed in the polygonally-shaped supporting cells of the crista ampullaris (**A, D'**- an empty white arrow). Myo7A-positive hair cells are separated by intervening CXCL1-positive supporting cells. DARC-positive cells are scattered within the crista's epithelium, with predominant localization in the central zone of each hemicrista **(B)**, sometimes surrounded by CXCL1-positive supporting cells (a solid white arrow) **(D')**. Scale bars, 100 μm **(A–D)**, 25 μm **(D')**. The pictures represent maximum projections of confocal z-stacks through whole mounts, based on biological replicates in 4 control cochleae.

Within the peripheral vestibular system, specific immunoreactivity for DARC was found within the crista ampullaris (*N* = 4 animals Figure 5B). Costaining for Myo7A, a hair cell marker (Figure 5C), and CXCL1 (Figure 5A) revealed that DARC was preferentially expressed in a subset of hair cells while CXCL1 was preferentially expressed in the surrounding supporting cells (Figure 5D).

## Discussion

### First Direct Measurements of Cytokines in Perilymph

To the best of our knowledge, this study provides the first direct measurements of cytokines in murine perilymph. The finding that the cytokine profile of the first one μL of fluid collected through the murine inner ear is different than the cytokine profile of the rest of the fluid collected through the PSC is consistent with the first microliter representing perilymph and the other fluid being related to CSF. This difference is expected based on a widely patent cochlear aqueduct in rodents, which connects CSF space with the inner ear (34). While CSF collected through the inner ear is similar to the CSF collected through the cisterna magna, these fluids are not identical in their cytokine profiles. The elevated cytokine levels in CSF collected through the inner ear but not through the cisterna magna after PTS-inducing noise exposure are most likely due to contamination by secretions or shedding from inner ear cells. Taken together, labyrinthine CSF is a proxy for but is not identical to the CSF bathing the brain.

Our measurements of proinflammatory cytokine levels in perilymph following acoustic trauma are in line with the previous measurements of similar cytokines in cochlear tissues. Specifically, elevation in TNF-α and IL-6 mRNA expression was reported in the stria vascularis, spiral ganglion neurons, and spiral ligament in the early phase after PTS-inducing acoustic trauma (124 dB SPL) by RT-PCR in Sprague-Dawley rats (18). In a follow-up study using identical noise parameters, the same group reported IL-6 protein upregulation in cochlear tissue lysates using western blot 6 h after acoustic trauma in C57BL/6J mice (21). Treatment with an anti-IL-6 receptor antibody after noise trauma ameliorated hearing loss (21).

When comparing mouse strains with different susceptibility to noise-induced hearing loss, several immune-related genes were reported to be upregulated in the susceptible mice (35). In addition, *Tnip1*, a gene encoding the “tumor necrosis factor, alpha-induced protein 3” was expressed at higher levels in mice resistant to noise exposure. This protein is known to inhibit TNF-mediated apoptosis and NF-kappa B activation.

Furthermore, supporting our results, ELISA of bulk cochlear tissue lysates following middle ear inoculation with *Haemophilus influenzae* to model acute otitis media (16) revealed cytokine levels in cochlear tissue that were generally within the same order of magnitude as the cytokine levels that we measured in cochlear fluid. However, the precise absolute values differed, at least in part due to differences in techniques, mouse strains, animal age and experimental models. Notably, IL-1β, IL-6, and CXCL1 levels were elevated in both studies while the levels of several cytokines analyzed in our study were below the detection limits of the assay used by Trune et al. (15) (e.g., IL-4, IL-5, IL-12, IFN-γ) (16). Together, these studies highlight the importance of proinflammatory cytokines in mediating different cochlear pathologies.

Complementing our results in the auditory periphery, a recent study demonstrated that neuroinflammation in the rodent auditory cortex following noise-induced hearing loss is associated with elevated tissue expression of proinflammatory cytokines, including TNF-α (36). Using genetic knockout and pharmacologic blockade of TNF-α, the authors conclude that targeting neuroinflammation may be a therapeutic strategy for tinnitus and other hearing loss-related disorders.

### CXCL1 and DARC in Cochlear Physiology and Pathology

While this is the first detailed description of the cellular localization of CXCL1 (formerly known as KC or Gro-α) within the mammalian cochlea, prior studies that analyzed pooled inner ear tissue noted upregulation of CXCL1 mRNA during cochlear inflammation and development. Specifically, CXCL1 mRNA was upregulated in the inner ear tissue of mice with chronic otitis media, based on qRT-PCR analysis (37). Further, CXCL1 and IL-6 mRNA expression was upregulated, based on qRT-PCR, in the cochlear lateral wall during inflammation following the pharmacological inhibition of the cochlear mitochondrial respiratory chain in a rat model (19). Together with TNF-α, CXCL1 mRNA expression was also upregulated in inner ear tissue after cochlear implantation in guinea pigs (38). Finally, the bulk RNA-Seq data from the developing organ of Corti revealed CXCL1 gene expression in “surrounding cells” (i.e., the collectively analyzed non-hair cells) (39). In other tissues, CXCL1 expression was reported in neutrophils, macrophages, and epithelial cells (40, 41).

Our finding of CXCL1 expression in the pillar cells, Deiters cells, interdental cells, and vestibular supporting cells of control animals suggests constitutive CXCL1 expression in the inner ear under physiologic conditions, and implicates these cell types in immune and inflammatory regulation in the inner ear. Our finding of reduced CXCL1 expression in pillar cells coupled with the elevated CXCL1 levels in perilymph after acoustic trauma suggests CXCL1 release into perilymph after pathologic noise exposure that damages pillar cells. The noise-induced elevation of CXCL1 levels in perilymph could also reflect higher production in the cochlear microenvironment. Since macrophages did not express CXCL1 6 h after acoustic trauma, the CXCL1-positive supporting cells appear to be the main producers of CXCL1. Relevantly, both pillar and Deiters cells express transforming growth factor-β-activated kinase-1 (TAK1) (42), a potent activator of proinflammatory signaling pathways including p38 (43), which is necessary for the activation of CXCL1 gene expression (44). Because the main activators of TAK1 are IL-1β and TNF-α (45), it is possible that these cytokines, in part produced by activated macrophages after noise trauma, can initiate TAK1-mediated activation of p38 and the consequent upregulation of CXCL1 gene expression in supporting cells. This process could take place very early after noise-induced trauma, since we found upregulation on CXCL1 gene expression as early as 2 h after PTS-inducing acoustic trauma (Supplementary Figure 5).

**Figure 6:**
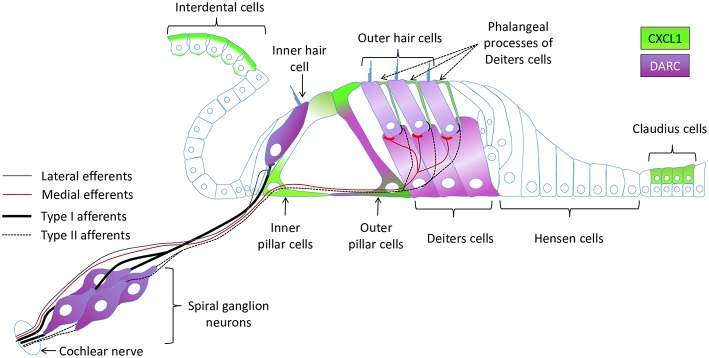
A schematic of the cochlear epithelium summarizing expression of CXCL1 and DARC within the organ of Corti and surrounding cells.

Apart from its proinflammatory activity, CXCL1 may play a neuromodulatory role in the inner ear given the close proximity of nerve fibers within the organ of Corti and CXCL1-expressing supporting cells (Figure 6). This hypothesis is inspired by studies in the central nervous system where CXCL1 has been implicated in the development and maintenance of neuropathic pain (46), as well as in promoting axon myelination (47) and triggering the resolution of tissue injury (48). By binding to chemokine receptor CXCR2 (49) expressed by neurons, CXCL1 may change neuronal membrane properties by modulating the expression and activity of ion channels.

While we did not detect CXCR2 expression in the cochlea, we describe for the first time cell-specific expression of another CXCL1 receptor, DARC, in the inner ear. Though DARC is well-known as an erythrocyte antigen, acting as a receptor for malarial parasites (*Plasmodium vivax* and *Plasmodium knowlesi*), its primary function is the sequestration and transcytosis of chemokines (50). Together with other atypical chemokine receptors (ACKRs), DARC (ACKR1) has the ability to bind different CC, and CXC chemokines, including CXCL1, which DARC binds with the highest affinity (51). In addition to erythrocytes, DARC is predominantly expressed on venular endothelial cells of various tissues (52), and on some specialized cell types such as renal epithelial cells (53), Purkinje cells (54), and bone marrow macrophages (55). Here we report that DARC is expressed in the SGNs and inner ear sensorineural epithelium, primarily in the inner hair cells, outer pillar cells, and the third row of Deiters cells. While the precise physiological role of DARC in the inner ear remains to be determined, a recent study of *Darc* knockout mice reported that DARC deficiency affords improved hearing recovery after intense noise exposure when compared to wild type mice (56). However, we did not detect a striking difference in the immunostaining intensity for cellular distribution of DARC in unexposed vs. noise-exposed animals. Moreover, our finding that CXCL1 and DARC are expressed in the sensorineural epithelium and spiral ganglion neurons under steady state conditions suggests a non-inflammatory role of CXCL1 and DARC in cochlear physiology. Furthermore, the juxtaposition of the pillar cells and the inner hair cells as the two cell types brightly positive for CXCL1 and DARC, respectively, suggests that CXCL1/DARC interaction may be involved in their communication. Although DARC has been assumed not to mediate G protein-coupled receptor (GPCR) signaling upon chemokine binding (50), GPCR-independent activation of the ERK1/2 MAPK pathway was reported in airway smooth muscle cells after CXCL1/DARC interaction (57). It is interesting that the viability of outer hair cells is tightly influenced by ERK1/2 phosphorylation status in Deiters cells (58), which we find to express CXCL1 and DARC. Additionally, the expression of DARC in outer pillar cells, which abundantly express CXCL1 in their apical and basilar segments, implicates the transcytosis and sequestration of CXCL1 in pillar cells as a possible role of DARC.

### CXCL1 as a Candidate Molecular Biomarker of Acute PTS

Our data suggest that CXCL1, potentially together with IL-6 and TNF-α, is a candidate molecular biomarker of acute PTS. The interplay between CXCL1 and IL-6 is known in other areas of the body, including upregulation of CXCL1 by IL-6 in the CNS (59), pancreatic acinar cells (60), and lung endothelium (61). TNF-α is also known to induce a marked release of CXCL1 in various cell types, including human vascular endothelial cells (62). Correspondingly, intraperitoneal injection of CXCL1 in mice induces TNF-α production (63). Moreover, TNF-α usually acts synergistically with IL-6 to enhance IL-6 secretion in human airway smooth muscle cells (64). However, IL-6 can elicit not only pro-, but also anti-inflammatory responses (65) and can increase the release of soluble TNF-α receptors (66).

### Inflammaging

Our studies in young compared to old animals revealed different patterns of age-related cytokine changes in perilymph and blood. In contrast to blood, where almost all tested proinflammatory cytokines were upregulated in old animals, IL-1β and TNF-α levels were significantly decreased in the perilymph of control animals. The detected elevation of IL-1β, IL-6, and TNF-α in the blood of older animals is in line with “inflammaging,” a phenomenon describing an age-related low-grade persistent increase in inflammatory molecules (67). However, the reduced levels of IL-1β and TNF-α in the perilymph of 2-year-old mice compared to adolescent mice suggests specialized mechanisms that regulate cytokine homeostasis in the inner ear with age. While Shi et al. (68) reported increased levels of IL-1β, IL-6, TNF- α, and IL-18 in the cochlear tissue of 1-year old mice compared to “young mice,” the age of “young mice” is not specified and their “old” mice were half the age of our old mice. Moreover, Shi et al. (68) did not specify mouse strain, making it difficult to directly compare the two studies (68). Another explanation for our findings is that there is loss of sensorineural and other cells in aging cochleae, resulting in fewer cells capable of releasing the detected cytokines.

Importantly, there is a correlation between cytokines and age-related hearing loss. A cohort study of 8,675 Japanese subjects revealed an association between hearing impairment and polymorphisms of genes encoding inflammatory mediators TNF-α and TNF receptor super family 1B (15). In mice, when comparing animals at 3 weeks and 3 months of age, increased expression of genes involved in the immune response (e.g., complement component 4, parts of immunoglobulins or *Ifit1*, interferon-induced protein with tetratricopeptide repeats) was noted at 3 months (69).

### Translational Implications

Our data lend further support to the idea that cytokines represent potential therapeutic targets for several inner ear maladies, including noise-induced hearing loss (19). Small molecules targeting TNF-α have already been used in clinical trials to ameliorate different types of (SNHL) [reviewed by (70)]. Similarly, small molecules targeting IL-6 and CXCL1 pathways are currently being used in clinical trials for non-otologic diseases. Our data strongly motivate future studies to test the repurposing of these small molecules to combat noise-induced hearing loss. Clearly, those repurposing studies will have to be carefully designed to consider the complexities of cytokine hemostasis, as illustrated by a recent clinical observation that a TNF-α reduction and an IL-6 increase in serum strongly correlated with a good therapeutic response after corticosteroid therapy for idiopathic sudden SNHL (71). Interestingly, noise induces not only changes in levels of perilymphatic molecules but also in perilymphatic volume (72), suggesting additional targets for future clinical therapies.

Our demonstration of the feasibility of detecting noise-induced and age-related changes in protein profiles in as little as 0.5 μL of perilymph opens future possibilities for liquid biopsy of the human inner ear based on perilymph sampling. Given that the volume of human perilymph is about 150-fold larger than the volume of mouse perilymph, clinical translation of our approach is within reach, with a negligible risk of hearing loss due to sampling of sub-μL volume of perilymph. Eventual routine liquid biopsy of inner ear fluid would address a major unmet medical need, and usher in an era of accurate molecular diagnosis of SNHL to guide precise, personalized therapies.

## Materials and Methods

### Mice

Wild-type CBA/CaJ mice were used because they have good cochlear sensitivity through most of their life. Mice (purchased from Jackson Laboratory, Bar Harbor, ME) of either sex were used in an estimated 50/50 ratio. They were randomly assigned to different noise exposure groups or to the control group at 6 weeks of age. Animal weight was comparable in all groups. Experiments were designed to typically have one mouse from each group housed in any given cage. When animals were not used for one of the interventions, they were kept in our animal care facility with *ad libitum* access to food and water. Ambient sound pressure levels in our animal care facility are in general under 40 dB SPL and peak noise levels do not exceed 70 dB SPL (73). A total of 100 mice were used to study the short- and long-term effects of noise exposure on cytokine levels in perilymph. Immunohistochemistry was performed on 4 control mice and 6 mice exposed to PTS-causing noise. All experiments were approved by the Institutional Animal Care and Use Committee at Massachusetts Eye and Ear.

### Noise Exposures (94, 97, and 103 dB Sound Pressure Level)

Experiments were carried out as described in the publication from our laboratory that established the noise levels that cause neuropathic TTS, non-neuropathic TTS and PTS in the cochlear base, specifically the areas where tones with frequencies of ~25 kHz and higher are processed (12). Briefly, mice were exposed for 2 h to an octave-band noise (8–16 kHz) at 94, 97, or 103 dB sound pressure level (SPL). Audio trauma was induced in the morning and the noise levels were calibrated with a microphone at the beginning of each session. The rodents were awake and could move freely in small cages under a horn in the exposure booth. Jensen et al. and other studies (12, 74) robustly and reproducibly documented the short and long-term effects of these noise levels on cochlear physiology and histology using the same equipment and mice of the same age and strain that we used here. Therefore, we did not replicate measurements of auditory function in the current study to avoid the potentially unfavorable effects of prolonged anesthesia (especially for the oldest animals) required to complete hearing tests prior to perilymph sampling.

### Perilymph Collection

For perilymph sampling we used the approach developed by Salt's and Hirose's laboratories (34). Briefly, mice were anesthetized with ketamine (100 mg/kg i.p.) and xylazine (10 mg/kg i.p.); half of the initial dose was given as booster. After fixing the head in a stereotaxic frame (David Kopf Instruments, Tujunga, CA), a retroauricular incision was made. Using blunt dissection of the subcutaneous tissue and underlying muscles, the PSC was exposed. The area was dried with absorbent points (Kerr, Orange, CA) and a layer of cyanoacrylate glue (Permabond 101, Permabond, Pottstown, PA) was applied to seal the area to obtain an uncontaminated sample. After the glue had dried, a 30° House oval window pick (Bausch+Lomb, Rochester, NY) was used to perforate the layer of glue and then make a canalostomy into the PSC. As soon as perilymph started to flow through the canalostomy, a calibrated (5 μL) disposable micropipette (VWR, Radnor, PA) was placed on top of it (Supplementary Figure 6 shows a schematic of perilymph sampling). The micropipettes had previously been marked to macroscopically indicate an estimate of the required sampling volume (0.5 μL for vestibular perilymph; 0.5 μL for cochlear perilymph; up to 5 μL for both types of CSF). Once the mark on the micropipette was reached, a 1 cm reticle with micrometer resolution (Carl Zeiss, Oberkochen, Germany) was used to quantify the exact volume of sampled perilymph under microscopic guidance. Because there is no physical separation of these types of liquid in the inner ear, the distinction between them is based on meticulous collection and previous calculations by the Salt and Hirose laboratories. Only clear specimens were processed further; samples that were contaminated with blood were discarded. Using a tube with a syringe (included when buying the microcapillary pipettes) the sample was transferred into an Eppendorf tube (Eppendorf, Hamburg, Germany) that had been prefilled with 10 μL of double-filtered PBS (Millipore Sigma, Burlington, MA). The tube was sealed with parafilm (Bemis Company, Neenah, WI), immediately placed on ice and transferred into a −80°C freezer until further use. Usually, the left ear was processed first. Once finished, the canalostomy in the PSC was covered with glue, and the right ear was sampled next.

### Cerebrospinal Fluid Collection

After bilateral specimen collection from the PSCs, CSF was collected from the cisterna magna as previously described (75). Briefly, the same disposable micropipettes as for perilymph sampling were pulled on a Sutter P-87 micropipette puller (Sutter instrument, Novato, CA) and trimmed with scissors to obtain a tip with an inner diameter of about 0.5 mm. A sagittal skin incision inferior to the occiput was made and subcutaneous tissue and muscles were separated. The skull over the dura magna was dried and the dura was penetrated with the microcapillary tube, which resulted in CSF filling the tube. The CSF specimen was then processed like the perilymph samples described above and stored in the −80°C freezer.

### Blood Collection

As the final procedure for every animal, a blood sample was obtained by cardiac puncture after thoracotomy. The sample was transferred into a serum gel with a clotting activator tube (Sarstedt, Nümbrecht, Germany) and left standing upright at room temperature for 30 min. It was then spun down (10,000 g for 5 min) and the supernatant was transferred into an Eppendorf tube that was sealed with parafilm and stored at −80°C.

### Immunoassay Based on Electrochemiluminescence

The V-PLEX Proinflammatory Panel 1 Mouse Kit (#K15048D, Meso Scale Discovery = MSD, Rockville, MD) was used to analyze the perilymph, CSF, and blood samples. It is an immunoassay based on electrochemiluminescence that quantifies the levels of the following cytokines: IFN-γ, IL-1β, IL-2, IL-4, IL-5, IL-6, CXCL1, IL-10, IL-12p70, and TNF-α. Briefly, samples and corresponding standards (calibrators) were added into wells of MSD MULTI-SPOT 96-well plates, pre-coated with capture antibodies, and incubated for 2 h at room temperature. Plates were washed three times with 150 μL/well of washing buffer, and 25 μL of detection antibody solution containing antibody conjugated with electrochemiluminescent labels (MSD SULFO-TAG) was applied into each well. Samples were incubated for an additional two h, then washed as described before, and treated with 2X Read Buffer T. Electrochemiluminescence was measured on the matching MSD SI2400 instrument. Concentration of cytokines was determined from electrochemiluminescence signals fitted to the calibration curve.

### Whole Mount Preparation and Confocal Immunohistochemistry

Cochlear whole mounts were dissected and mounted as described previously (76). Immunofluorescent staining was carried out as reported (77). The following antibodies/stains at the specified concentrations were used: rabbit anti-Myosin 7A (Myo7A, #25-6790 Proteus Biosciences, Ramona, CA, 1:400), mouse anti-Myosin 7A (Myo7A, #138-1-S, Developmental Studies Hybridoma Bank, Iowa City, IA, 1:10), rabbit anti-GRO alpha (CXCL1) biotin (#NBP1-51188B, Novus Biologicals, Littleton, CO, 1:150), mouse anti-E-Cadherin (#610181 BD Biosciences, San Jose, CA, 1:200), sheep-anti DARC (#PA5-47861 Thermo Fisher Scientific, Waltham, MA, 1:25) Phalloidin (#A22283 Thermo Fisher Scientific, Waltham, MA, 1:200), Alexa Fluor 555 anti-mouse and Alexa Fluor 647 anti-rabbit (#A21422 and #A21245 Thermo Fisher Scientific, Waltham, MA, 1:1,000), Alexa Fluor 488 anti-sheep (#A-11015 Thermo Fisher Scientific, Waltham, MA, 1:400), Streptavidin, Alexa Fluor 488 (#S11223, Thermo Fisher Scientific, Waltham, MA, 1:200). For the quantification of inner and outer hair cell numbers before and after noise exposure, the same confocal microscopy settings were used (Leica SP5, Wetzlar, Germany). Images were obtained with 20x, 40x, and 63x objectives. The z-step size was 1.5–2 μm, and cells were counted over a distance of 300 μm.

### Statistical Analysis

GraphPad PRISM (GraphPad Software Inc., La Jolla, CA) was used for all statistical analyses. For cytokine measurements, Bartlett's test was used to check for homoscedasticity. When variances among the groups were equal, one-way ANOVA was used to compare the groups. If ANOVA was significant (*p* < 0.05), pair-wise comparison was performed and Bonferroni correction was applied for multiple comparisons. When variances were not equal, Kruskal-Wallis test was used. If the test was significant (*p* < 0.05), Wilcoxon rank-sum test was applied, followed by Bonferroni correction. Composite graphs showing individual and average values were constructed using GraphPad PRISM.

## Data Availability

All datasets generated for this study are included in the manuscript/Supplementary Files.

## Ethics Statement

The animal study was reviewed and approved by Institutional Animal Care and Use Committee at Massachusetts Eye and Ear.

## Author Contributions

KMS conceived the project, designed experiments, and supervised all aspects of research. Perilymph samples were collected by LDL, TF, and VYS. Noise exposures were carried out by LDL and RS. Dissection of whole mounts was performed by LDL, SV, and RS. Immunohistochemical staining, confocal microscopy, and image analysis was performed by SV. Perilymph samples were processed by LDL, SV, and LX. TF performed statistical analyses of perilymph samples. LDL, SV, and KMS wrote the manuscript with editorial input from all other authors.

### Conflict of Interest Statement

The authors declare that the research was conducted in the absence of any commercial or financial relationships that could be construed as a potential conflict of interest.
