# Supplementary material for: Cytokine Levels in Inner Ear Fluid of Young and Aged Mice as Molecular Biomarkers of Noise-Induced Hearing Loss
Source: Front Neurol. 2019 Sep 11;10:977. doi: 10.3389/fneur.2019.00977 (PMC6749100; doi:10.3389/fneur.2019.00977)
Supplement: Supplementary file 1 [file Data_Sheet_1.docx]

# Supplementary Data

**Paraffin embedding, immunohistochemistry, and light microscopy**

All mice processed for paraffin sections and whole mounts were sacrificed by transcardiac perfusion. Cochleae were extracted, fixed in 4% paraformaldehyde for 1 h, decalcified in 0.12 M EDTA for 48 h, embedded in paraffin and cut in 10 μm-thick serial sections in a plane parallel to the midmodiolar axis using a Leica RM 2155 microtome. Using a standard protocol that we have previously published (1), sections were deparaffinized with xylene, washed in 100% ethanol and rehydrated through a series of graded ethanols. Immunohistochemistry was performed using anti-GRO alpha (CXCL1) biotin (#NBP1-51188B, Novus Biologicals, Littleton, CO, 1:150) and anti-Iba1 (#019-19741, Wako Pure Chemical Industries, Ltd., Osaka, Japan, 1:2500) primary antibodies, followed by rabbit AlexaFluor 488 Tyramide SuperBoost Kit Goat anti-rabbit IgG (#B40943, Thermo Fisher Scientific, Waltham, MA) and Streptavidin, Alexa Fluor® 488 (#S11223, Thermo Fisher Scientific, Waltham, MA, 1:200) secondary antibodies.

**Quantitative RT-PCR**

The microdissection of cochleae and isolation of cochlear soft tissue was performed in glass-bottom dishes filled with RNAlater reagent (#7020, Ambion, Inc., Austin, TX) as described previously (2). Total RNA was isolated using the RNeasy® Mini Kit (50) (#74104, Qiagen, Germantown, MD) and treated with RNase-Free DNase Set (#79254, Qiagen, Germantown, MD). cDNA was synthesized using the SuperScript IV First-Strand Synthesis System (#18091050, Thermo Fisher Scientific, Waltham, MA) and random hexamers. Real-time PCR was performed by QuantStudio 3 real-time PCR system (#A28136, Applied Biosystems, Waltham, MA) using TaqMan Fast Advanced Master Mix (#4444556) and TaqMan probes: CXCL1 (#Mm04207460_m1) and 18S rRNA (#Hs99999901_s1) (Thermo Fisher Scientific, Waltham, MA). The following parameters for 96-well plates were used: 50°C for 2 minutes, 95°C for 10 minutes, and 45 cycles of 95°C for 15 seconds followed by 60°C for 1 minute. Cochlear samples were run in triplicates. Relative fold changes in mRNA levels were calculated after normalization to 18S rRNA using 2−ΔΔCT method.

**Statistical analysis**

For cytocochleogram analysis (quantification of hair cell numbers in control vs exposed mice), unpaired t test with Welch's correction was used.

# Supplementary Figures and Tables

**Supplementary Figure 1. Fold change of cytokine levels in murine perilymph and cerebrospinal fluid six hours after noise exposure.** Six-week-old mice were exposed to 8–16 kHz noise for 2 hours at 94 dB SPL, 97 dB SPL, and 103 dB SPL; unexposed mice served as controls (CTRL). Six hours post exposure, vestibular perilymph (vPLF), cochlear perilymph (cPLF), and cerebrospinal fluid (CSF) were collected through the posterior semicircular canal. In addition, CSF was collected via cisterna magna (cmCSF). Each dot represents fold change level normalized to cytokine values from unexposed mice. Data are shown as group means + standard error of the mean. N=16-20 ears for vPLF and cPLF, each; N=14-17 ears for CSF; N=7-9 ears for cmCSF; N=10-19 ears for control samples. P < 0.05 (*), < 0.01 (**) or < 0.001 (***).

**Supplementary Figure 2. Levels of Th-related cytokines (IFN-γ, IL-2, IL-4, IL-5) and immunoregulatory cytokine (IL-10 and IL-12) in murine perilymph, cerebrospinal fluid, and blood six hours, two weeks, and two years after noise exposure.** Six-week-old mice were exposed to 8–16 kHz noise for 2 hours at 94 dB SPL and 97 dB SPL; unexposed mice served as controls. Samples were collected six hours, two weeks, and two years post exposure. Vestibular perilymph (vPLF), cochlear perilymph (cPLF), cerebrospinal fluid (CSF), and blood were collected as described in Fig. 3. Each dot represents measurements from one ear (vPLF, cPLF and CSF) or animal (blood). Data are shown as group means + standard error of the mean. N=3-20 ears for vPLF; N=3-20 ears for cPLF; N=3-17 ears for CSF; N=4-10 animals for blood; N=7-10 ears for control samples. P < 0.05 (*), < 0.01 (**) or < 0.001 (***). NT-not tested.

**Supplementary Figure 3.** **Cytocochleograms of hair cells.** Whole mounts of control mice and age-matched mice exposed to 8–16 kHz noise for 2 hours at 103 dB SPL noise at 6 weeks of age and sacrificed 6 hours later were stained with anti-Myo7A antibodies, and cells were manually counted using confocal microscopy. Myo7A-positive outer hair cells (OHCs) (A) and Myo7A-positive inner hair cells (IHCs) (B) were manually quantified as mean +/- SEM (n= 3 or 5 different cochleae) per 100 µm of cochlear length in whole mounts (as exemplified in Figure 5B). A significant loss of OHCs was observed in the basal and mid-basal region of noise-exposed animals; the number of IHCs was not significantly changed. The gray vertical bar depicts the noise band. Individual symbols represent individual cochleae *p < 0.05, **p < 0.01, ***p < 0.001.

**Supplementary Figure 4. Macrophages are not the main source of CXCL1 expression in the murine cochlea.** Fluorescence microscopy of paraffin-embedded cochlear cross-sections in control mice (A) or age-matched mice exposed to PTS (103 dB SPL) at 6 weeks of age, and sacrificed 6 hours later (B, C) revealed immunostaining for CXCL1 in the pillar cells of the organ of Corti (asterisk) (A, B). Iba1-positive macrophages located within the spiral ligament (white arrowheads) and spiral limbus did not express CXCL1 irrespective of noise exposure (C). Representative images are from the cochlear base, based on biological replicates in 3 control and 3 noise-exposed animals. Scale bar, 100 µm.

**Supplementary Figure 5.** Time- dependent upregulation of *Cxcl1* gene expression in noise-exposed animals. Six-week-old mice were exposed to 8–16 kHz noise for 2 hours at 103 dB SPL; unexposed mice served as controls. Cochlear tissue was collected one, two, and three hours post exposure. A significant upregulation of *Cxcl1* gene expression is manifested as early as 2 hours after PTS-inducing acoustic trauma. Each dot represents measurements from one ear (N=4 control ears, N=4 noise-exposed ears). Data are shown as means ± standard error of the mean. P < 0.05 (*).

**Supplementary Figure 6. Schematic of murine perilymph sampling.** Sampling through an opening in the posterior semicircular canal using a microcapillary pipette. Close-up image in the upper right corner demonstrates the flow diagram of inner ear fluids during sampling (as described by Hirose et al., 2014). CSF entering through the cochlear aqueduct displaces perilymph and causes a flow through the entire cochlea and the vestibule to exit at the semicircular canal.

**Supplementary Table 1.** **CXCL1 expression in the cochlea.**

| **Cell types** | **Control** | | | **103 dB noise** | |
| --- | --- | --- | --- | --- | --- |
|  | **Apical Turn** | | **Basal Turn** | **Apical Turn** | **Basal Turn** |
| Claudius cells | | +++ | +++ | +++ | +++ |
| Deiters cells (row 1-2) | | ++ | +/++ | + | +/++ |
| Outer pillar cells | | +/++ | -/+ | ++ | -/+ |
| Pillar cells (apical part) | | +++ | ++/+++ | +++ | +/++ |
| Inner pillar cells | | ++ | + | ++ | + |
| Interdental cells | | +++ | +++ | +++ | +++ |

Expression level: +++ bright; ++ moderate; + low; - negative

**Supplementary Table 2.** **DARC expression in the cochlea.**

| **Cell types** | **Control** | | | **103 dB noise** | |
| --- | --- | --- | --- | --- | --- |
|  | **Apical Turn** | | **Basal Turn** | **Apical Turn** | **Basal Turn** |
| Claudius cells | | -/+ | + | +/++ | + |
| Deiters cells (row 3) | | -/+ | + | +/++ | + |
| Deiters cells (row 1-2) | | -/+ | -/+ | + | + |
| Outer hair cells | | +/++ | +/++ | +/++ | + |
| Outer pillar cells | | + | -/+ | + | -/+ |
| Inner pillar cells | | -/+ | - | -/+ | - |
| Inner hair cells | | ++ | +++ | ++ | ++ |
| Spiral ganglion neurons | | ++ | ++ | +++ | ++ |

Expression level: +++ bright; ++ moderate; + low; - negative

# References

[1] S.Y. Kao, J.S. Kempfle, J.B. Jensen, D. Perez-Fernandez, A.C. Lysaght, A.S. Edge, and K.M. Stankovic, Loss of osteoprotegerin expression in the inner ear causes degeneration of the cochlear nerve and sensorineural hearing loss. Neurobiology of disease 56 (2013) 25-33.

[2] K.M. Stankovic and G. Corfas. Real-time quantitative RT-PCR for low-abundance transcripts in the inner ear: analysis of neurotrophic factor expression. Hearing Research. (2003). 185:97–108.
