# Supplementary figures and images for: Cytokine Levels in Inner Ear Fluid of Young and Aged Mice as Molecular Biomarkers of Noise-Induced Hearing Loss
Source: Front Neurol. 2019 Sep 11;10:977. doi: 10.3389/fneur.2019.00977 (PMC6749100; doi:10.3389/fneur.2019.00977)

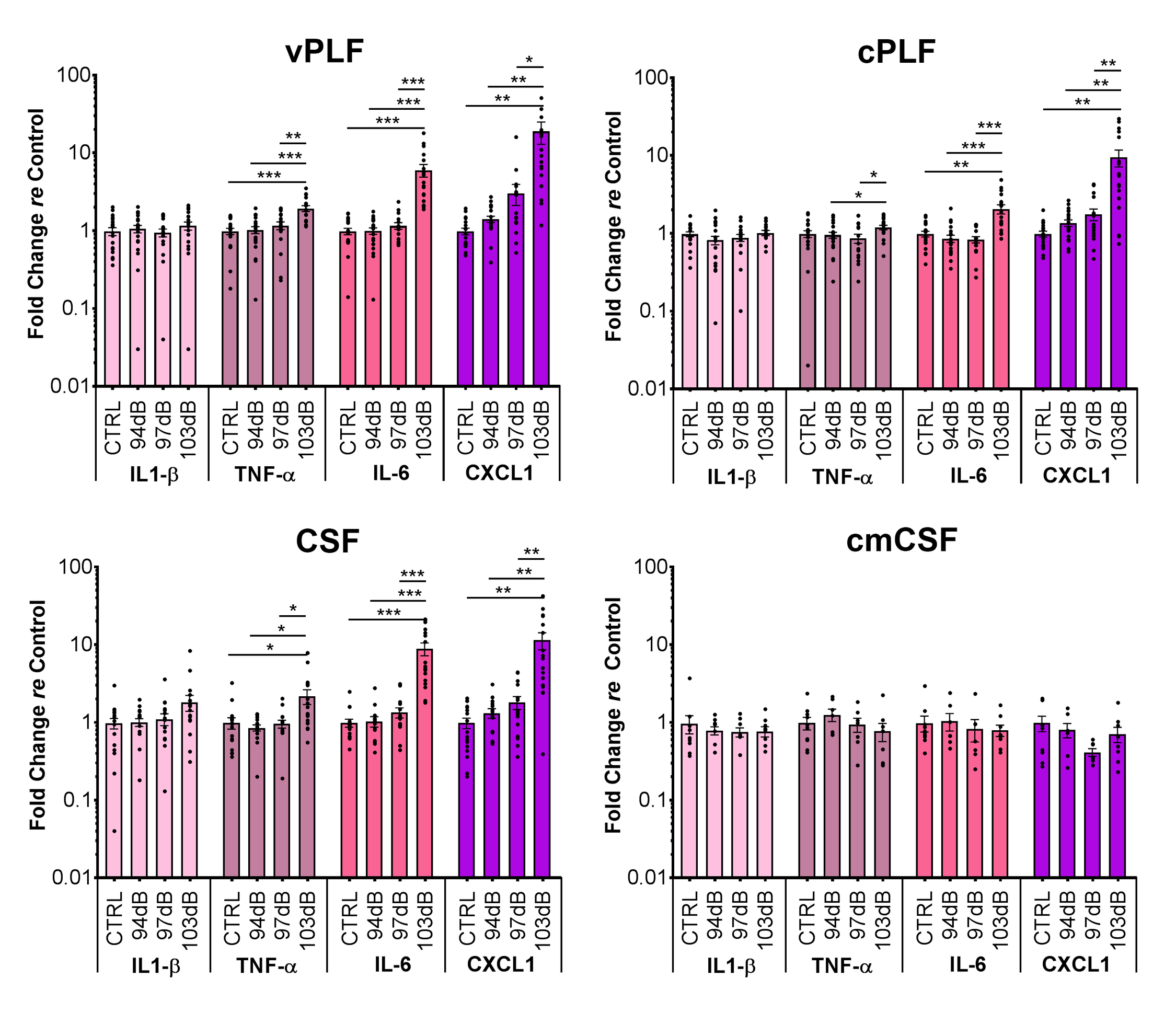

Supplement: Supplementary file 2 [file Image_1.tif]

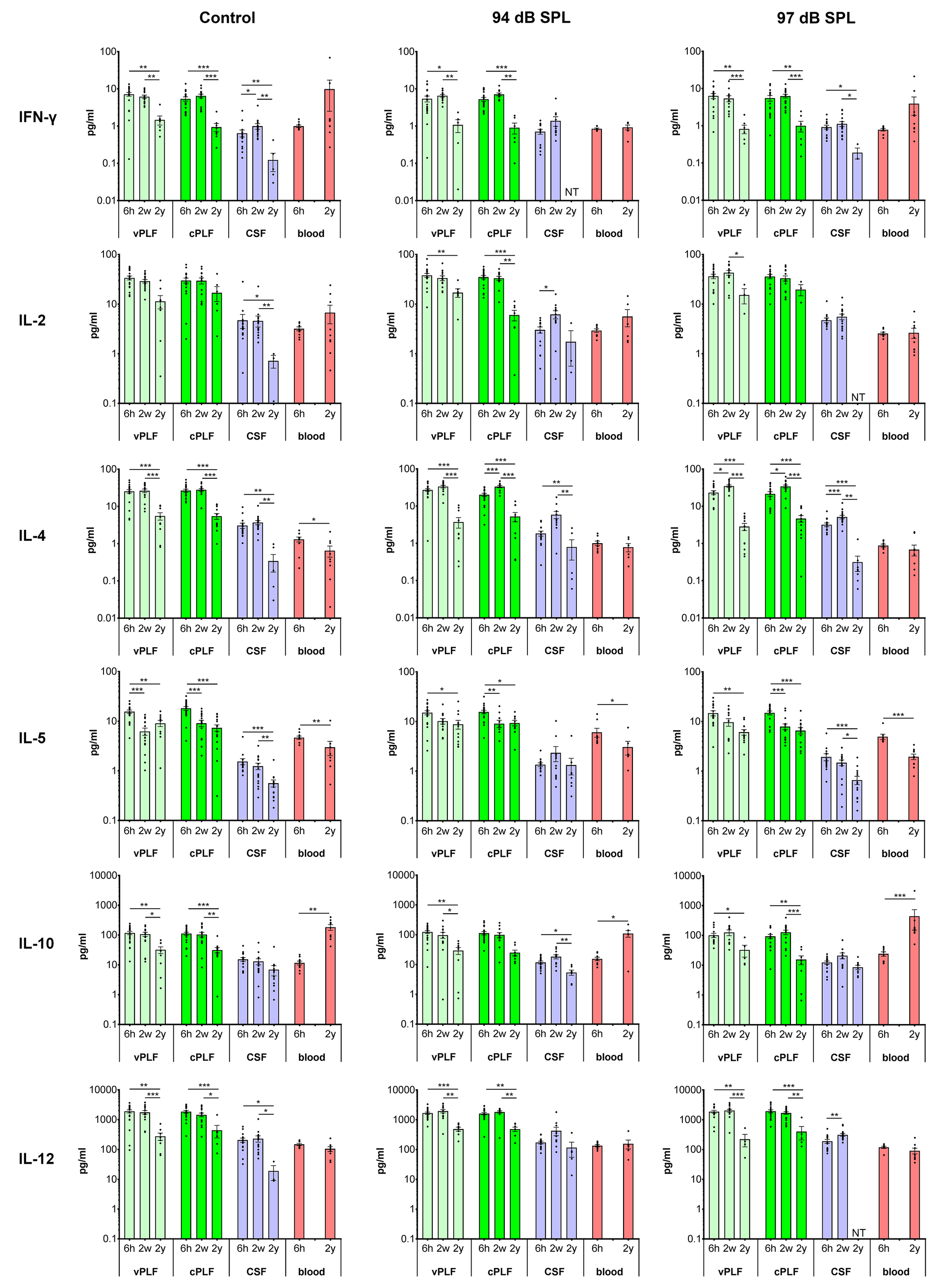

Supplement: Supplementary file 3 [file Image_2.TIF]

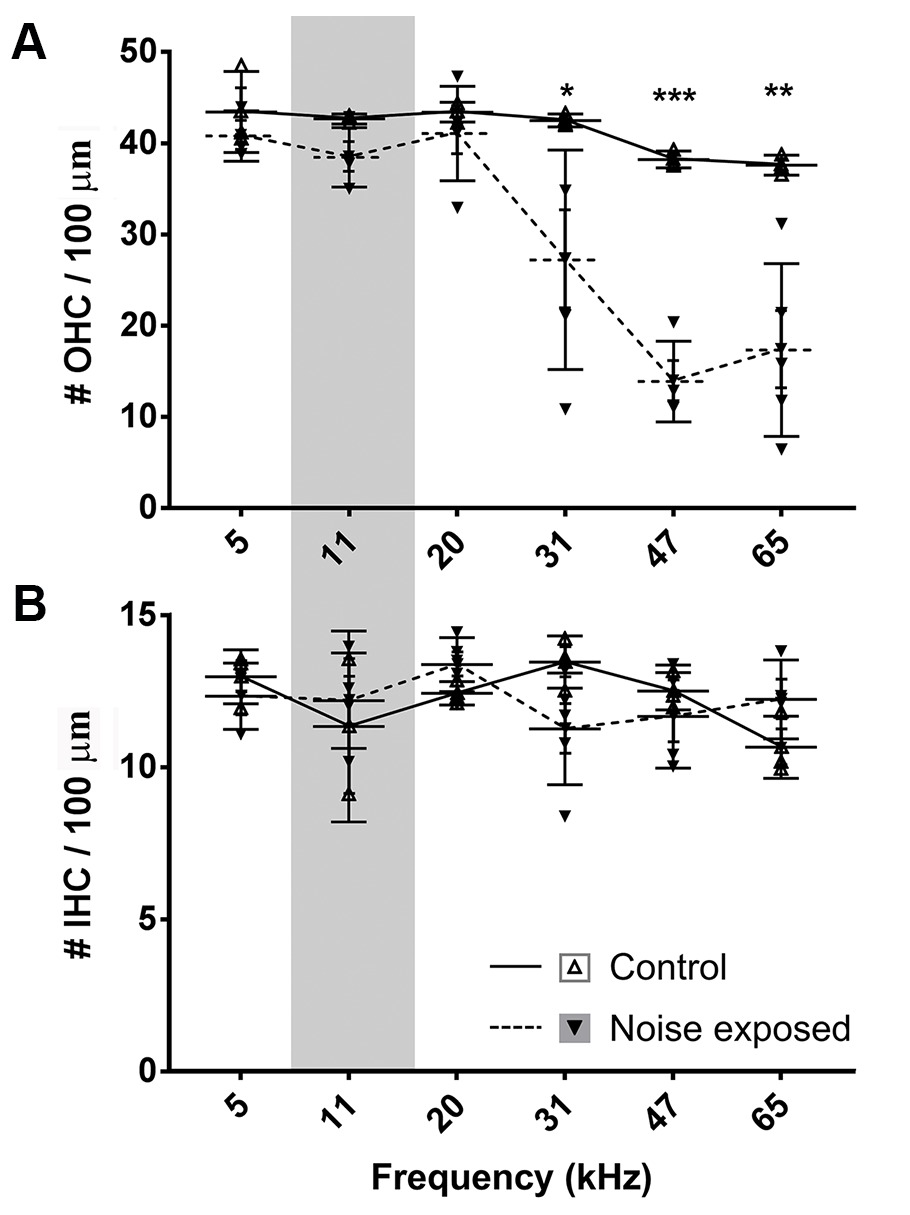

Supplement: Supplementary file 4 [file Image_3.TIF]

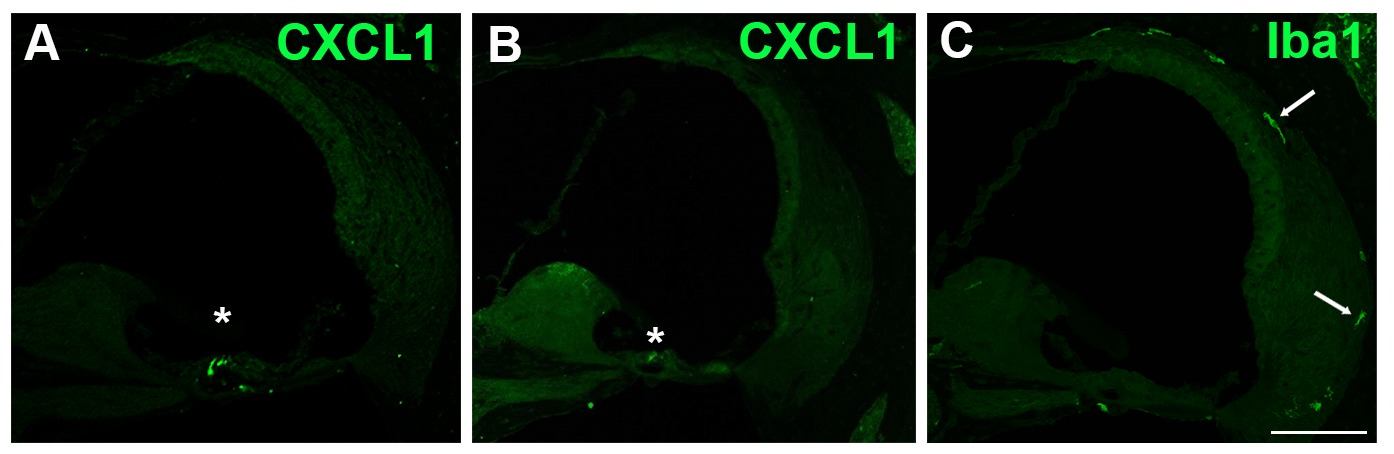

Supplement: Supplementary file 5 [file Image_4.TIF]

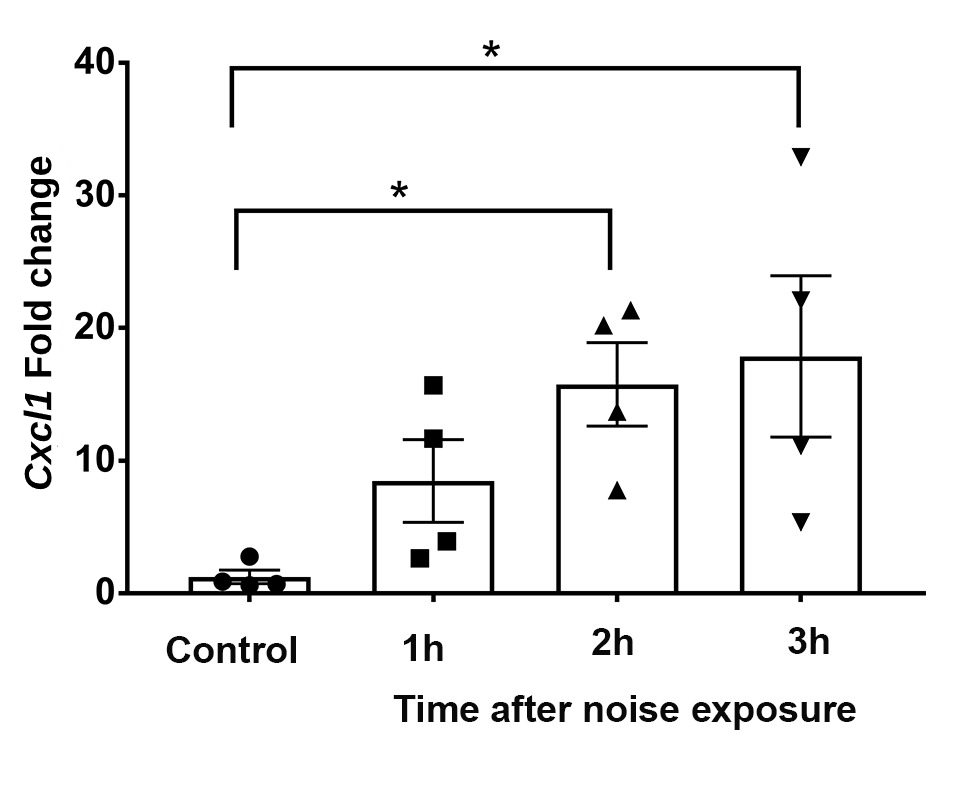

Supplement: Supplementary file 6 [file Image_5.TIF]

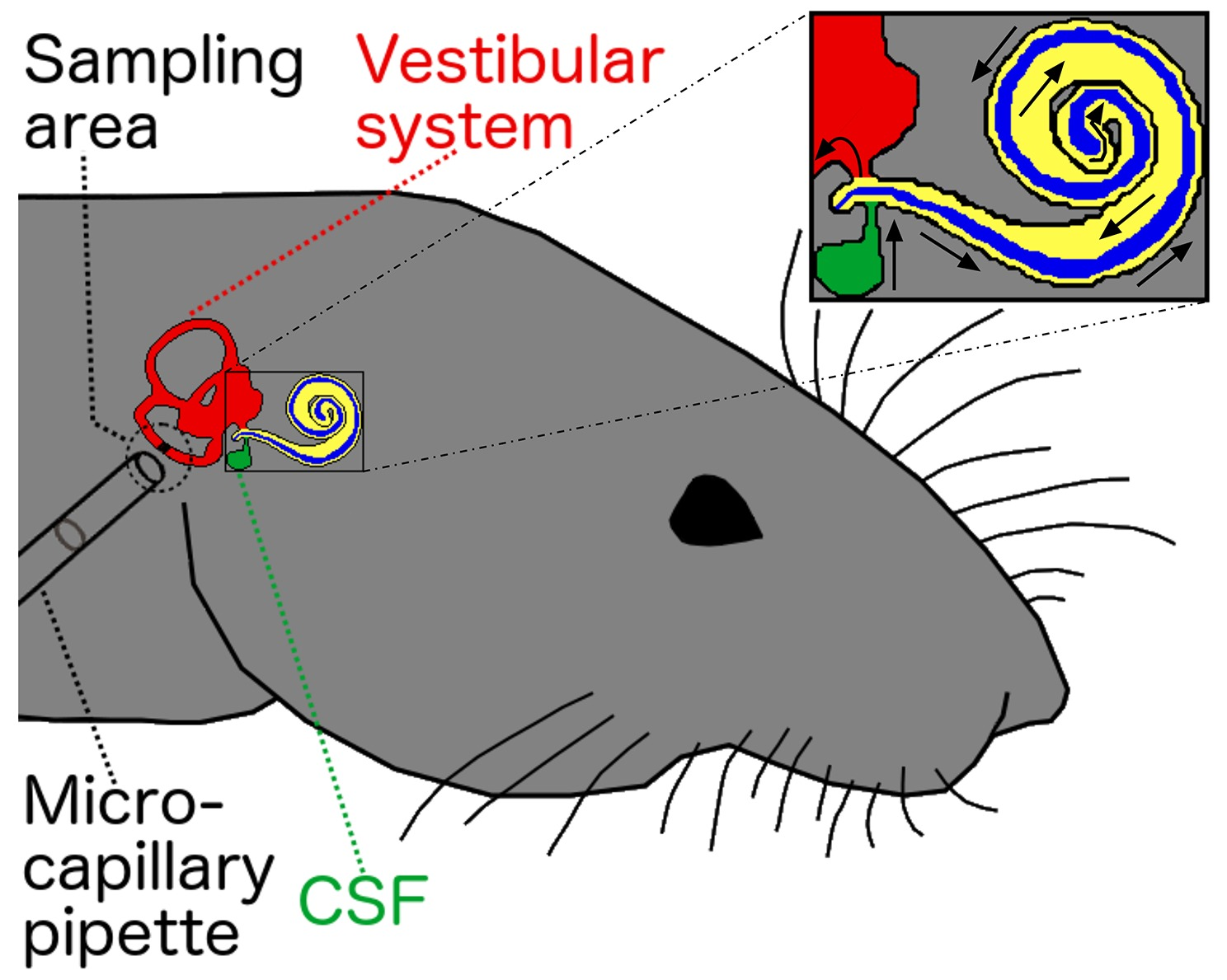

Supplement: Supplementary file 7 [file Image_6.TIF]
